# A Novel Use of a Not So “EZ-Blocker” For Lung Isolation During a Direct Transaortic Transcatheter Aortic Valve Replacement

**DOI:** 10.7759/cureus.58110

**Published:** 2024-04-12

**Authors:** Kenneth John, Becca Berube, Danielle Sawka, Shyamal R Asher

**Affiliations:** 1 Anesthesiology, Beth Israel Deaconess Medical Center, Harvard Medical School, Boston, USA; 2 Anesthesiology, The Warren Alpert Medical School of Brown University, Providence, USA; 3 Anesthesiology, Rhode Island Hospital, Providence, USA

**Keywords:** transcatheter aortic valve replacement (tavr), cardiac anesthesia, ez blocker, lung isolation, direct transaortic tavr, tavr( transcatheter aortic valve replacement)

## Abstract

Alternate access transcatheter aortic valve replacement presents unique challenges for anesthesiologists, including the possible need for lung isolation while working with space constraints around the patient's airway. Troubleshooting lung isolation in these cases can be challenging, requiring quick thinking and adaptability while maintaining patient safety. We present a case of direct transaortic transcatheter aortic valve replacement with an endobronchial blocker ("EZ-blocker") used for lung isolation that required a novel use of the "EZ-blocker" to achieve adequate lung isolation.

## Introduction

Transcatheter aortic valve replacement (TAVR) is a minimally invasive procedure that has become an increasingly popular treatment approach for aortic valve disease, such as aortic stenosis (AS) [1]. TAVRs have been shown to have comparable symptom improvement and all-cause mortality to surgical aortic valve replacement (SAVR) in high-risk populations [2].

Patient risk stratification is essential when considering TAVR procedural approach, which includes coronary artery assessment, pulmonary function tests, and carotid dopplers. Patients also undergo a CT scan of the chest, abdomen, and pelvis to measure the aortic annulus for prosthetic valve sizing and to determine procedure access routes [3]. The most common vascular access is transfemoral. However, for approximately 10% of patients, this route is not an option due to small femoral arterial size, tortuous arteries, excess calcification, and atherosclerotic disease of the aorta [4]. Other approaches to TAVR include transcarotid, suprasternal, subclavian, direct aortic, transcaval, and transapical access.

This report presents the case of a patient with symptomatic and severe AS who underwent a TAVR requiring a direct transaortic approach via right anterior thoracotomy. Intraoperatively, lung isolation was challenging, requiring a novel use of an endobronchial blocker ("EZ-blocker" [EZB]). This case report will also discuss the unique anesthetic considerations when managing patients with alternative access TAVRs.

## Case presentation

An 80-year-old female with a past medical history significant for hypertension, carotid stenosis, bifascicular heart block, and extensive peripheral vascular disease with severe cerebrovascular disease presented for treatment of symptomatic critical AS (aortic valve area of 0.7 cm^2^) with preserved left ventricular function.

She was determined to be at high risk for brain hypoperfusion during an SAVR due to her four-vessel cerebrovascular disease and was recommended for a TAVR. Given her moderate-to-severe iliofemoral disease on imaging, a direct aortic approach TAVR was determined to be the best approach for the patient.

**Figure 1:**
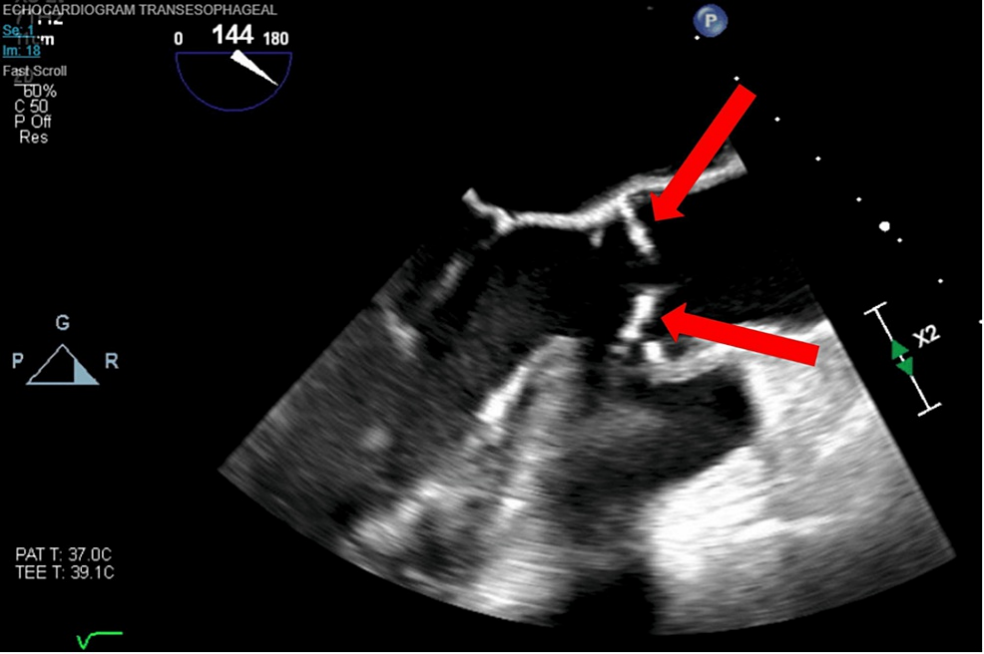
A transesophageal echocardiographic view of the aortic valve in the long axis showing thickened aortic valve leaflets with minimal opening leading to severe aortic stenosis (red arrows).

On the day of surgery, large-bore intravenous and arterial access were obtained in the preoperative holding area. Once in the operating room, general anesthesia was initiated with an 8.0 endotracheal tube (ETT) in place. A central venous multiple access catheter (MAC) was placed with a temporary pacer wire floated through the introducer for rapid pacing during valve deployment and due to her risk for permanent heart block. An EZB was placed under bronchoscopic visualization, and the right lung was isolated to allow for better surgical visualization of the aorta during the right anterior thoracotomy. Thereafter, a transesophageal echocardiography (TEE) probe was placed and used to perform a complete cardiac examination, which confirmed severe AS (Figure 1).

An anterior thoracotomy was performed by the cardiothoracic surgeon at the level of the right second and third rib space. Despite the EZB being in place with the right bronchial balloon inflated, the surgeon noted that the right lung would inflate at various times, crowding the surgical field and impairing visualization of the aorta. Each time lung isolation was lost, a bronchoscopic examination revealed that the EZB had advanced down the right main stem bronchus past the carina and wedged into the right upper lobe and bronchus intermedius. Attempts to correct the position of the EZB were shortlived as the EZB would migrate again, likely due to maneuvering of the TEE probe and/or the ETT. Interestingly, each time the EZB migrated, the surgeon noted that the right upper lobe remained deflated while the middle and lower lobes started reinflating.

**Figure 2:**
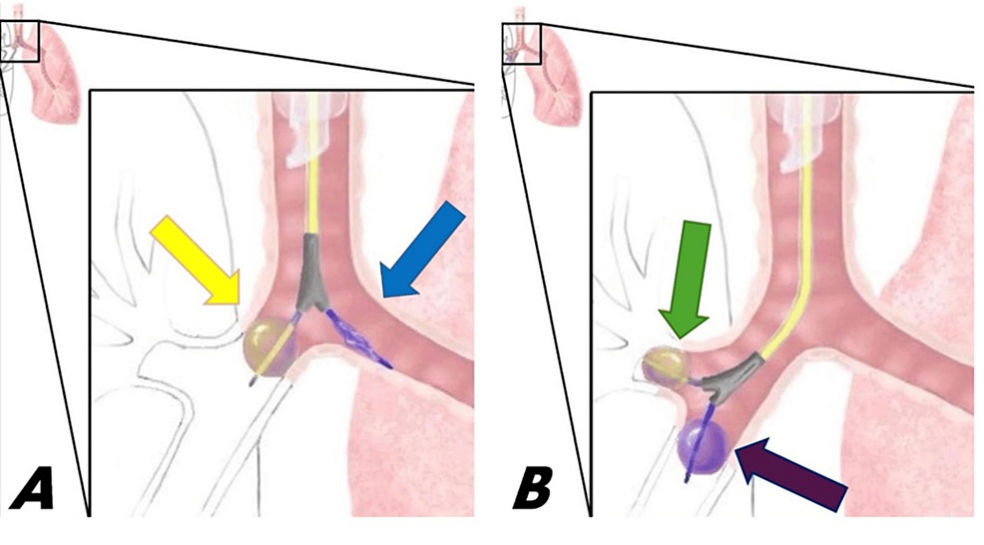
Use of an EZ blocker in the standard and novel positions A. This is an artistic demonstration of the ideal placement of the EZ-blocker sitting at the level of the carina, with the right cuff inflated (yellow arrow) to isolate ventilation only to the left lung (blue arrow). B. This represents the non-standard use of the EZ-blocker that was required in this case with the blocker at the secondary carina at the level of the right upper lobe (green arrow) and bronchus intermedius (purple arrow). Both cuffs were inflated to prevent ventilation of the right lung. Image designed and illustrated by D Sawka.

After multiple failed attempts at using the EZB in the standard fashion at the level of the carina to isolate the right lung, the decision was made to use the EZB in a unique and non-standard fashion. Under bronchoscopic guidance, the EZB was placed at the right secondary carina at the branch of the right upper lobe and bronchus intermedius. Thereafter, both EZB balloons were inflated simultaneously to allow for complete isolation of the right lung (Figure 2).

This maneuver allowed for adequate lung isolation and surgical exposure, thereby facilitating the progress of the case. The remainder of the procedure continued as planned without complications. At the end of the case, the EZB balloons were both deflated and the EZB was removed without any signs of trauma to the trachea or right mainstem bronchus. The patient ultimately was extubated uneventfully and was discharged on postoperative day 4.

## Discussion

TAVRs are most commonly performed with a transfemoral approach [5]. However, patient anatomy may require alternative approaches including direct aortic access, as required and performed in our patient. Alternate approach TAVRs are typically more procedurally complex with unique anesthetic implications. However, when alternative approaches are compared to transfemoral access TAVR, multiple studies have found no difference in mortality, lower rate of unplanned vascular repairs, lower major vascular complications, and no increased risk of stroke [6].

The anesthetic implications of direct aortic approach TAVRs include the need for general anesthesia, the need for adequate lung isolation if a thoracotomy approach is planned, and TEE guidance to evaluate the bioprosthetic aortic valve after deployment [3]. Lung isolation can be achieved with a double-lumen tube (DLT), a single-lumen ETT with a bronchial blocker, or a single-lumen endobronchial tube. In our case, we elected to use a single-lumen ETT with an EZB for lung isolation. A DLT was not selected due to its larger size, which may make placement and manipulation of a TEE probe more challenging in this patient. The EZB was initially placed appropriately at the level of the carina confirmed using bronchoscopy. However, the EZB was determined to migrate into the right main stem during the operation, disrupting the surgical exposure and progress. This was likely due to TEE probe manipulation resulting in ETT movement and EZB translocation.

Other management strategies were considered to improve lung isolation, including repositioning the single-lumen ETT, switching to a different bronchial blocker, or switching to a DLT. However, procedural and practical consideration of this case precluded us from pursuing these strategies. As the procedure had already commenced, the area around the patient’s airway was already crowded with the TEE machine and echocardiographer, fluoroscopy equipment, and surgical staff around the patient’s head. Troubleshooting the lung isolation with bronchoscopy was therefore suboptimal. Furthermore, the operation had progressed to a point where halting and clearing the space for airway manipulation was not an option.

Given these unique circumstances, we opted for a non-standard approach when using an EZB where we placed the blocker at the branch of the right upper lobe and bronchus intermedius with both balloons inflated simultaneously (Figure 2). To our knowledge, this approach has never been previously reported in the literature. The main risks considered with this novel approach included airway injury around the secondary carina due to the smaller diameters, and dislodgement of the EZB resulting in both left and right mainstem bronchi occlusion, preventing any ventilation. These risks were managed with limited cuff inflation pressures of the EZB and monitoring of the peak pressure alarms on the ventilator. This technique ultimately led to adequate lung isolation for the procedure to continue. No issues were encountered from this moment to the end of the procedure which concluded with the patient being extubated and transported to the postanesthesia care unit.

## Conclusions

Direct transaortic TAVRs present unique challenges to anesthetic care, including the need for TEE guidance during the surgery and possible need for lung isolation with limited access to the patient's airway intraoperatively. In this case, an EZB was used to achieve lung isolation but was ineffective while in the standard position due to migration into the right mainstem bronchus. Therefore, a novel use of the EZB was necessary at the level of the right secondary carina at the branch of the right upper lobe and bronchus intermedius with both balloons simultaneously inflated to isolate the right lung. We report that this novel position of an EZB is safe and effective. This case demonstrates the need for the anesthesia team to remain adaptable and think quickly while keeping patient safety as the paramount concern in these challenging cases.
